# Redescription of *Platynaspis flavoguttata* (Gorham) (Coleoptera, Coccinellidae) and notes on nomenclature of *Platynaspis kapuri* Chakraborty & Biswas

**DOI:** 10.3897/BDJ.2.e1096

**Published:** 2014-05-23

**Authors:** J. Poorani

**Affiliations:** † National Bureau of Agriculturally Important Insects, P.B. No. 2491, HA Farm Post, Bellary Road, Bangalore 560024, India

**Keywords:** Platynaspis flavoguttata, redescription, Platynaspis kapuri, Coccinellidae

## Abstract

*Platynaspis flavoguttata* (Gorham) (Coleoptera: Coccinellidae) is redescribed and the male genitalia are illustrated for the first time. It is also recorded from Sri Lanka for the first time. *Platynaspis bimaculata* (Hoang, 1983) is a new junior synonym of *Platynaspis bimaculata* Pang & Mao, 1979 (**new synonym**). *Platynaspis kapuri* Chakraborty & Biswas, 2000, the replacement name for *Platynaspis bimaculata* Pang & Mao, 1979 established by Ukrainsky (2007), is also the new replacement name for *Platynaspis bimaculata* (Hoang, 1983), as both are junior homonyms of *Platynaspis bimaculata* Weise, 1888 besides being synonyms. *Platynaspis hoangi* Ukrainsky (2007) is an unnecessary replacement name for *Platynaspis bimaculata* (Hoang).

## Introduction

The genus *Platynaspis* Redtenbacher 1844 (Coleoptera: Coccinellidae) is currently placed in the subfamily Coccinellinae as per the recent classification proposed for Coccinellidae by Seago et al. (2011). This genus is distributed in Africa, Madagascar and the Oriental region. In India, it is mainly confined to the northeastern and northwestern regions and only *Platynaspis flavoguttata* (Gorham 1894) has been hitherto known from peninsular India (Poorani 2002). Three Oriental genera, *Paraplatynaspis* Hoang, *Phymatosternus* Miyatake, and *Platynaspidius* Miyatake, were synonymized with *Platynaspis* by Slipinski and Tomaszewska (2002) as they considered them to constitute only specialized clades within *Platynaspis*. *Platynaspis* is represented by 11 species in the Indian subcontinent. *Platynaspis flavoguttata*, a rare species, was recently collected from the southern Indian state of Karnataka and is redescribed here and the male genitalia are illustrated for the first time to facilitate identification. Nomenclatural notes on *Platynaspis bimaculata* Pang and Mao (1979) and *Platynaspis bimaculata* (Hoang 1983) are provided.

## Materials and methods

Images of whole specimens and their diagnostic characters, including male genitalia, were generated using Leica M205A stereo microscope. Composite images from image stacks were generated using Combine ZP and touched up for clarity and resolution in Adobe Photoshop Elements 11. The specimens studied are housed in the reference collections of National Bureau of Agriculturally Important Insects, Bangalore, India.

## Taxon treatments

### Platynaspis flavoguttata

(Gorham, 1894)

Scymnus? flavoguttatus  Gorham 1894: 208 (BMNH).
Pharus flavoguttata : Weise 1895: 157.
Platynaspis flavoguttata : Sicard 1913: 501. – Korschefsky 1932:  232.

#### Materials

- **Type status:** Other material. **Occurrence:** recordedBy: A.N. Reddy; individualCount: 5; sex: 2 females, 1 male; **Location:** country: India; stateProvince: Karnataka; verbatimLocality: Sagara: Mulllumane; verbatimElevation: 589 m; verbatimLatitude: 14.33°N; verbatimLongitude: 74.79°E; **Event:** samplingProtocol: Sweep net; eventDate: 2012-11-24; **Record Level:** institutionCode: National Bureau of Agriculturally Important Insects (NBAII)
- **Type status:** Other material. **Occurrence:** recordedBy: H.J. Bremer; individualCount: 3; sex: 1 male, 2 females; **Location:** country: Sri Lanka; stateProvince: Southern Province: Galle District; verbatimLocality: Habaraduwa; **Event:** eventDate: 1982-08-20/1982-09-04; **Record Level:** institutionCode: National Bureau of Agriculturally Important Insects (NBAII)

#### Description

Form (Fig. 1) broad oval, moderately convex, densely pubescent with a mixture of yellow and dark brown hairs. Head luteous yellow with a longitudinal median reddish brown band or reddish brown with a pair of yellowish lateral spots or fully reddish brown, with a mixture of short, recumbent white hairs and much longer, suberect dark brown to black hairs. Pronotum dark reddish brown with three luteous yellow markings on posterior margin, median spot somewhat spindle-shaped, constricted towards both ends, lateral spots subtriangular; pubescence similar to head with a mixture of short, recumbent white hairs and long, suberect dark brown hairs. Each elytron with three spots in a 2-1 arrangement, first two spots positioned in anterior half just before middle, discal one transverse, not touching sutural line, lateral spot circular, touching lateral margin of elytron, posterior spot placed in apical 1/3 before apical margin, not touching lateral margin; pubescence with a mixture of short, yellowish recumbent hairs more or less confined to elytral spots, and a mixture of short, recumbent and much longer, dark brown, suberect hairs on darker areas of elytra. Ventral side reddish castaneous except antennae, mouthparts, and legs lighter yellowish brown, with yellowish white, recumbent pubescence, lateral margins of epipleura with dark brown erect hairs. Head (Fig. 1b) with clypeal margin deeply, semicircularly emarginate, punctures dual, with dark brown hairs arising out of slightly larger punctures, separated by 2–5 diameters. Pronotum with dual punctures similar to head, punctures denser, more closely placed than those on head. Elytra with dual punctures, punctures separated by 2–4 diameters, dark hairs arising from larger punctures. Epipleura foveolate on level of mid and hind legs to receive tibial apices. Abdomen with five ventrites, abdominal postcoxal lines on ventrite 1 as in *Diomus* Mulsant (Fig. 2a), short, extending posteriorly to hind margin and merged with hind margin of ventrite 1; posterior margin of ventrite 1 medially slightly concave. Posterior margin of ventrite 5 broadly arcuate in female, truncate in male. Male genitalia (Fig. 2b, c, d) as illustrated; tegmen in lateral view (Fig. 2b) with parameres much broader than penis guide, paddle-like, apically obliquely transverse, with elongate hairs; penis guide in inner view (Fig. 2c) lanceolate in outline, progressively broadened up to a little beyond middle, apical third triangular, gradually narrowed to a bluntly rounded apex; penis (Fig. 2d) with a prominent, broad basal capsule.

#### Diagnosis

This species has a distinctive dorsal colour pattern by which it can be differentiated from the other known Indian species of the genus. The male genitalia also are diagnostic.

#### Distribution

India (Karnataka), Myanmar (Korschefsky 1932, Poorani 2002); Sri Lanka (new distribution record).

#### Ecology

Not known.

#### Biology

Gorham (1894) observed the specimens of *Platynaspis flavoguttata* "living in amity with red ants in a hole in *Terminalia paniculata*".

#### Notes

The specimens examined from Sri Lanka (Fig. 1d) show some minor variations in the dorsal colour pattern and the size and shape of the elytral spots as follows: head more or less fully brown; pronotum reddish brown with subtriangular, luteous yellow lateral markings; elytron with the discal spot in the anterior half distinctly more rounded, apical spot more transverse and roughly crescent-shaped.

### Platynaspis kapuri

Chakraborti & Biswas, 2000

Platynaspis kapuri  Chakraborty and Biswas 2000: 122 (Holotype male, Zoological Survey of India, Kolkata).
Platynaspis bimaculata  Pang and Mao 1979: 94–95 (preoccupied in Weise 1888). – Ukrainsky 2007: 212.
Platynaspidius bimaculata  Poorani 2002: 315.
Paraplatynaspis bimaculatus  Hoang 1983: 8–9. – Slipinski and Tomaszewska 2002: 496 (synonymy with *Platynaspis*). – Ukrainsky 2007: 212. **New synonym.**
Platynaspis hoangi  Ukrainsky 2007: 212. Unnecessary replacement name for *Platynaspis bimaculata* (Hoang).

#### Materials

- **Type status:** Other material. **Occurrence:** recordedBy: Commonwealth Institute of Biological Control-Indian Station; sex: 4 females, 4 males; **Taxon:** acceptedNameUsage: Platynaspis kapuri; originalNameUsage: *Platynaspis bimaculata* Pang et Mao 1979; **Location:** continent: Asia; country: India; stateProvince: Assam; verbatimLocality: Hajo; **Identification:** identifiedBy: J. Poorani; **Event:** eventDate: 1965-12-12; **Record Level:** institutionCode: National Bureau of Agriculturally Important Insects (NBAII)

## Discussion

*Platynaspis bimaculata* (Hoang 1983), originally designated as the type of *Paraplatynaspis* Hoang (1983), is conspecific with *Platynaspis bimaculata* Pang and Mao (1979) as the habitus and male genitalia illustrations given by Hoang are identical to those of the Indian specimens studied (Figs 3, 4) (**new synonym**). Pang and Mao (1979) and Ren et al. (2009) also illustrated this species. Both *Platynaspis bimaculata* Pang & Mao and *Platynaspis bimaculata* (Hoang) are junior secondary homonyms of *Platynaspis bimaculata* Weise (1888) as pointed out by Ukrainsky (2007). Ukrainsky (2007) elevated *Platynaspis kapuri* Chakraborty and Biswas (2000), a junior synonym of *Platynaspis bimaculata* Pang & Mao (Poorani 2004), as a new replacement name for the latter and proposed a new replacement name, *Platynaspis hoangi* for Hoang’s species, as he was probably unaware that both *Platynaspis bimaculata* Pang & Mao and *Platynaspis bimaculata* Hoang are synonymous themselves. As per Article 60.2 of the International Code of Zoological Nomenclature (4th edition) on junior homonyms with synonyms, if the rejected junior homonym has one or more available and potentially valid synonyms, the oldest of these becomes the valid name of the taxon with its own authorship and date. Hence, *Platynaspis kapuri* Chakraborty and Biswas (2000), a junior synonym of *Platynaspis bimaculata* Pang & Mao, becomes the replacement name also for *Platynaspis bimaculata* Hoang as it is synonymous with *Platynaspis bimaculata* Pang & Mao (**stat. rev.**) and *Platynaspis hoangi* Ukrainsky is an unnecessary replacement name for *Platynaspis bimaculata* (Hoang). This species is distributed in northeastern India, Vietnam and China. It is externally quite variable (Fig. 3a, b, c) and the male genitalia (Fig. 4b, c) are diagnostic.

## Supplementary Material

XML Treatment for Platynaspis flavoguttata

XML Treatment for Platynaspis kapuri

## Figures and Tables

**Figure 1a.**
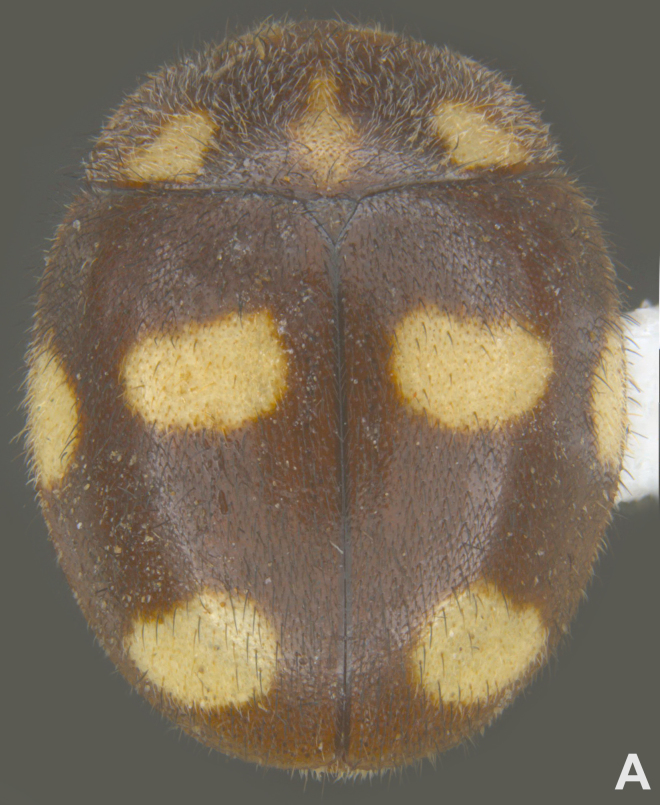
Dorsal view

**Figure 1b.**
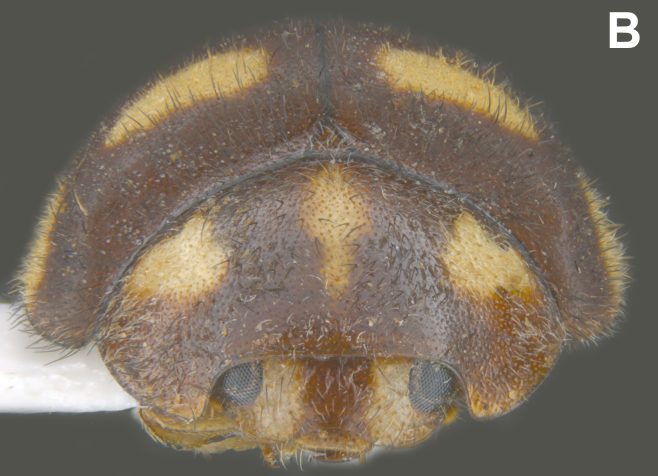
Frontal view

**Figure 1c.**
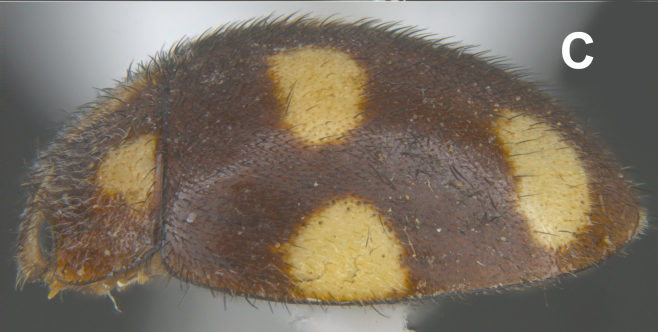
Lateral view

**Figure 1d.**
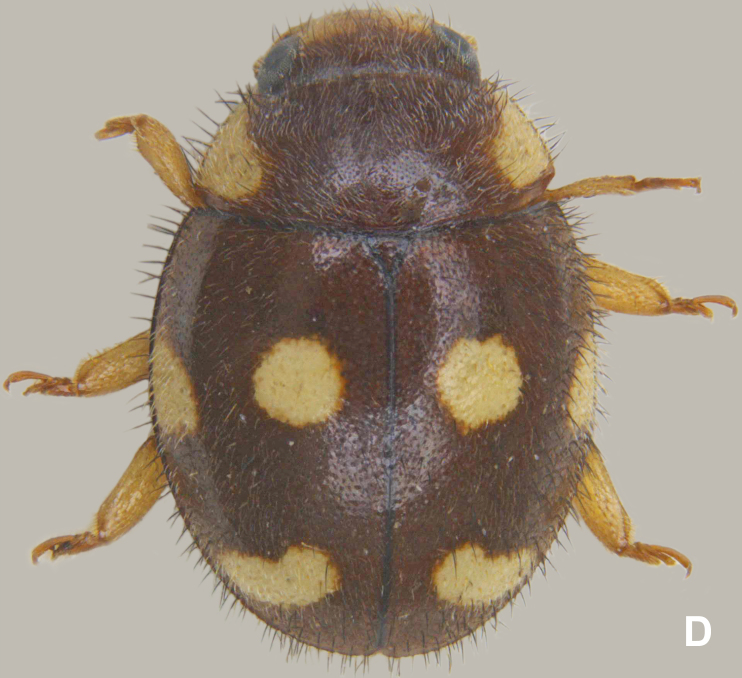
Form from Sri Lanka

**Figure 2a.**
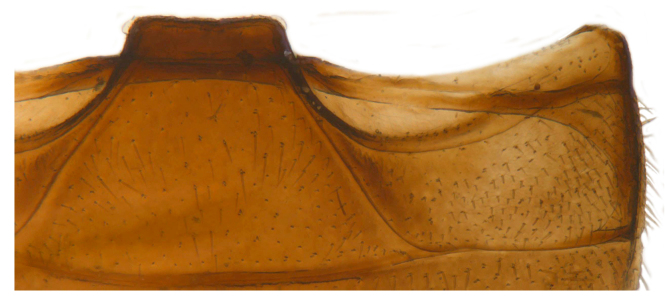
Abdominal postcoxal line

**Figure 2b.**
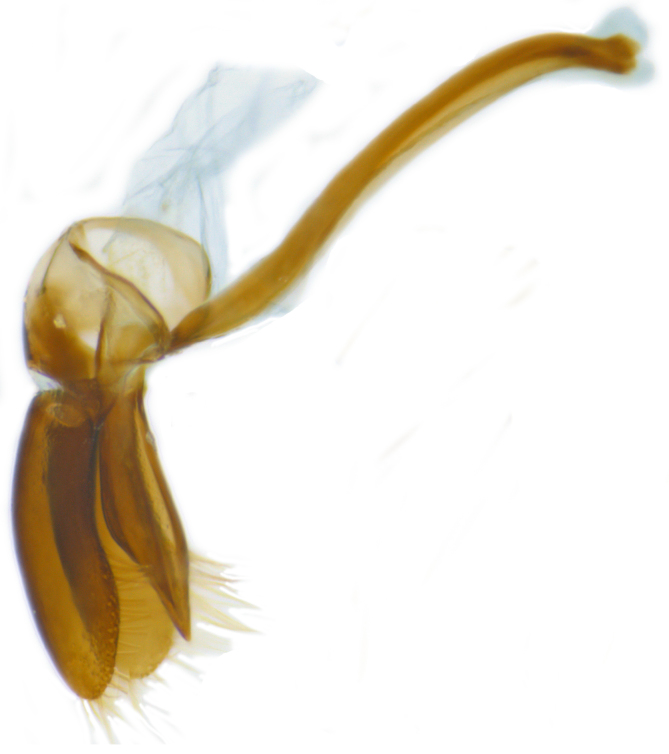
Male genitalia: Tegmen, lateral view

**Figure 2c.**
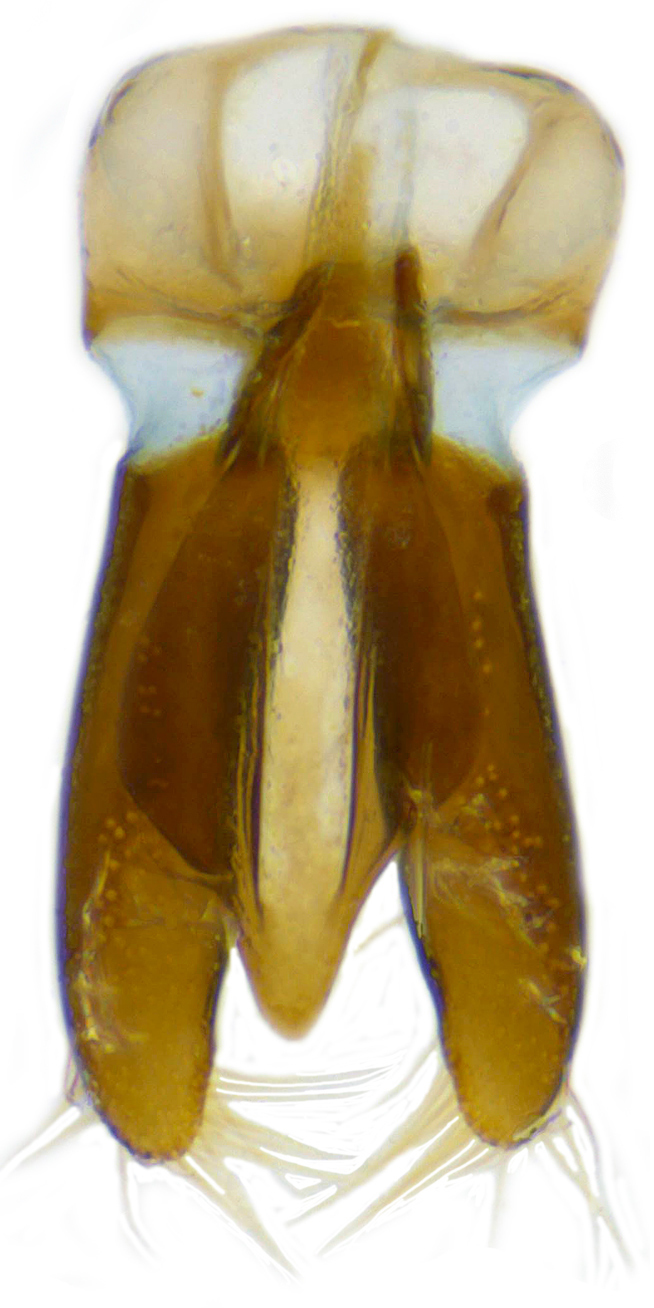
Male genitalia: Tegmen, inner view

**Figure 2d.**
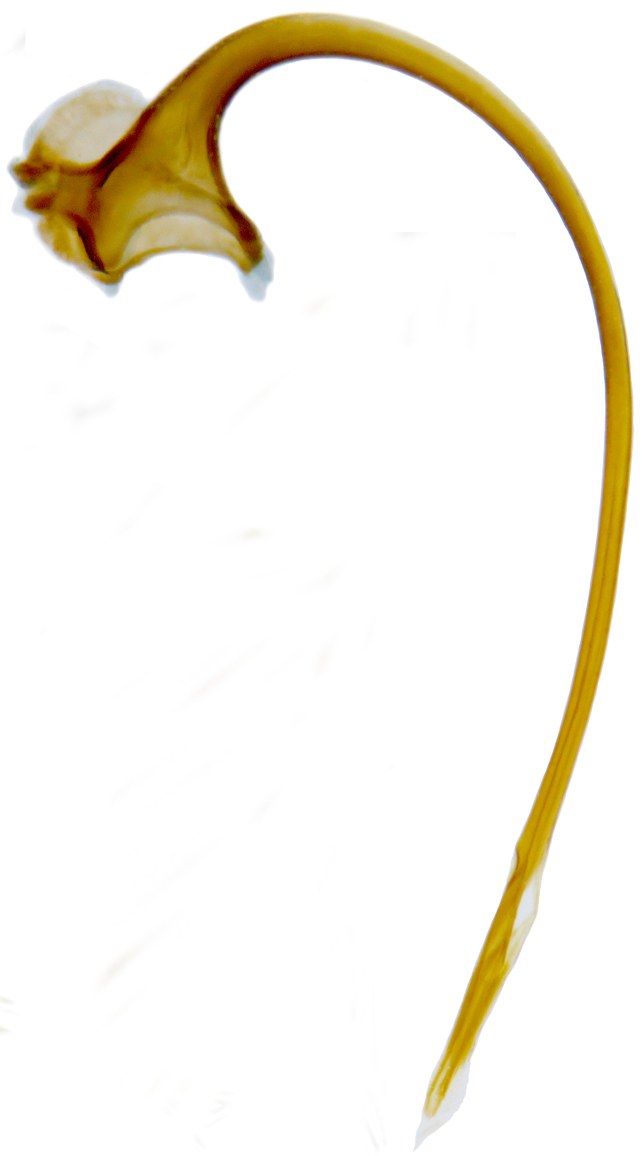
Male genitalia: Penis

**Figure 3a.**
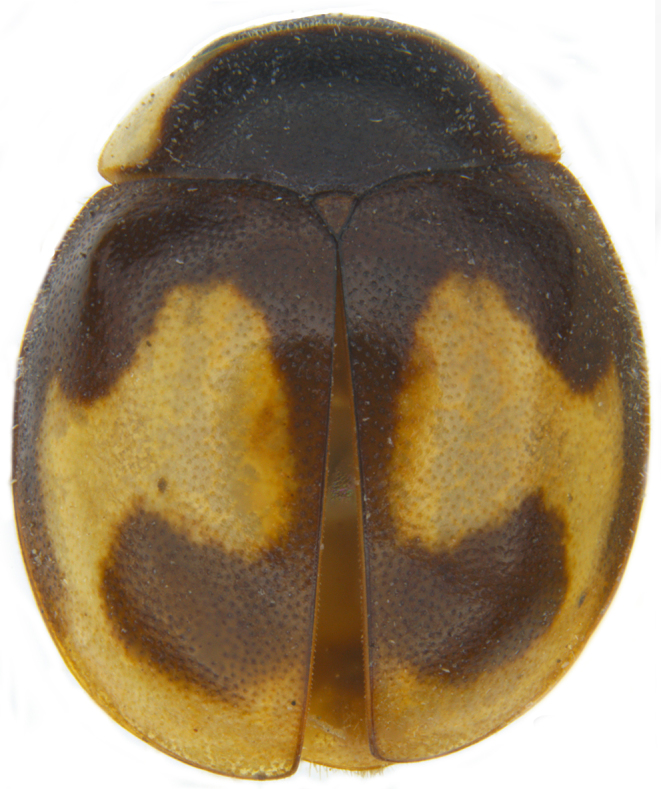
Common morph

**Figure 3b.**
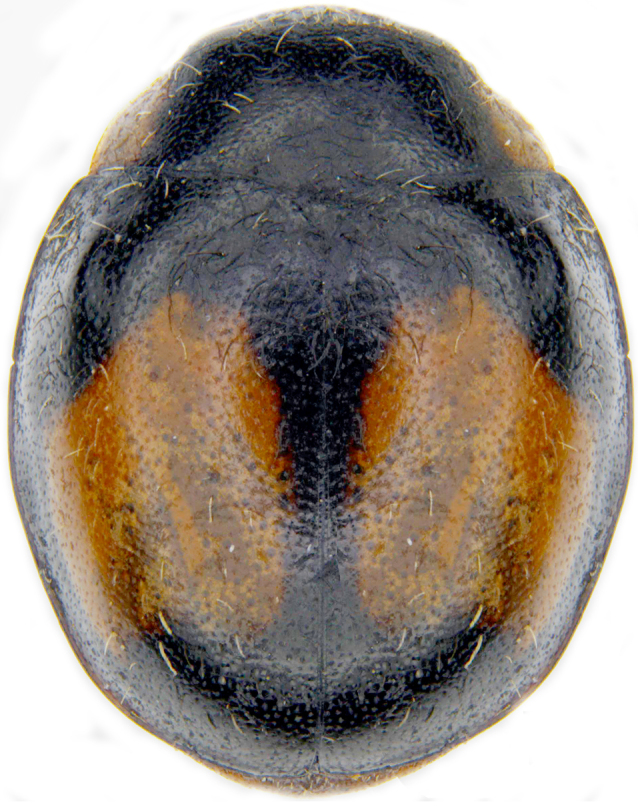
Variant

**Figure 3c.**
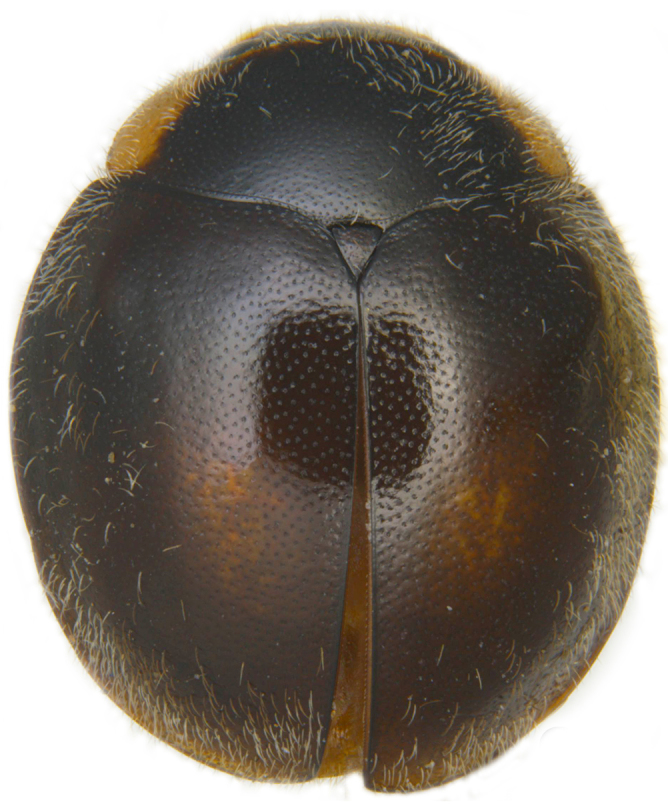
Form with black elytra

**Figure 4a.**
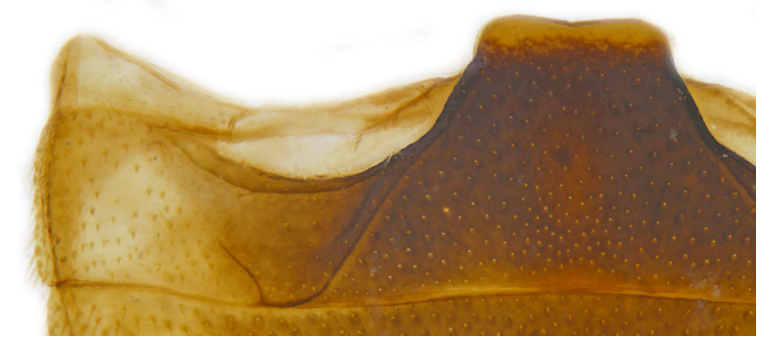
Abdominal postcoxal line

**Figure 4b.**
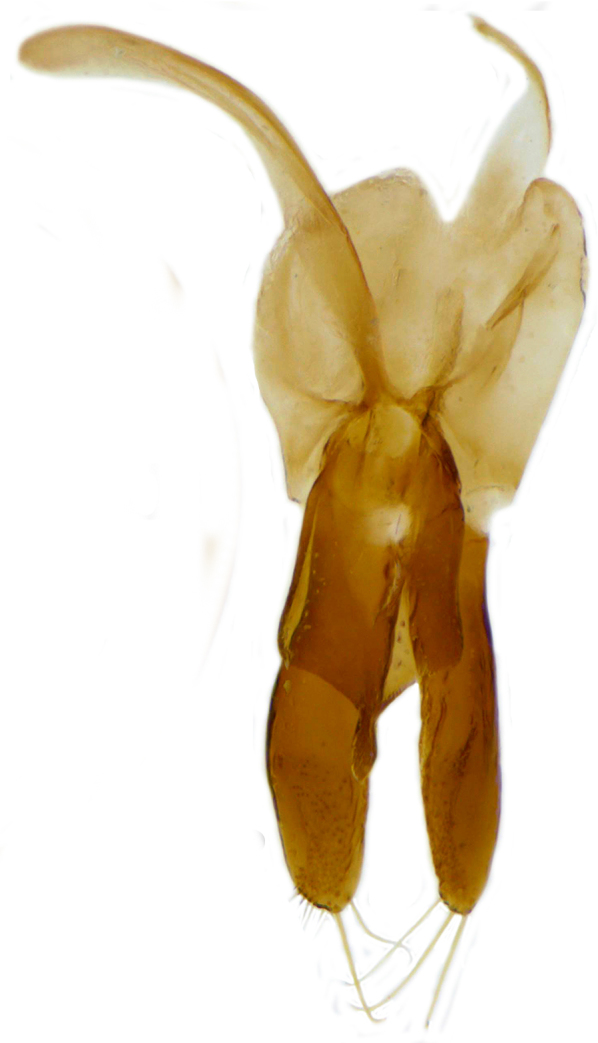
Male genitalia: Tegmen, inner view

**Figure 4c.**
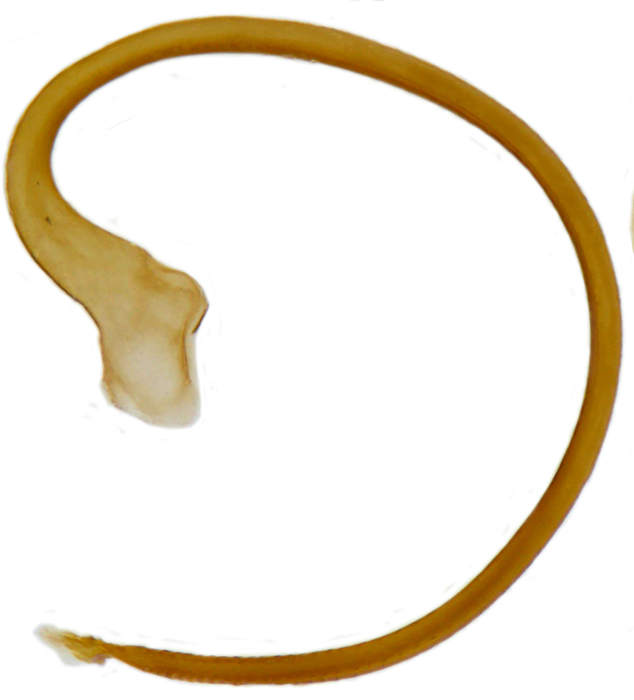
Male genitalia: Penis
